# Groundwater and human development: synergies and trade-offs within the context of the sustainable development goals

**DOI:** 10.1007/s11625-017-0490-9

**Published:** 2017-09-18

**Authors:** Maya Velis, Kirstin I. Conti, Frank Biermann

**Affiliations:** 1 Copernicus Institute of Sustainable Development, Utrecht University, Heidelberglaan 2, 3584 CS Utrecht, The Netherlands; 2 International Groundwater Resources Assessment Centre, Westvest 7, 2611 AX Delft, The Netherlands; 3 Governance and Inclusive Development, Amsterdam Institute of Social Sciences Research (AISSR), University of Amsterdam, Nieuwe Achtergracht 166, 1018 WV Amsterdam, The Netherlands; 4 Present Address: Ministry of Infrastructure and the Environment for the Kingdom of the Netherlands, 2515 XP The Hague, The Netherlands

**Keywords:** Groundwater, Sustainable development, Human development, Synergies, Trade-offs

## Abstract

This article argues that groundwater—accounting for 98% of all fresh water on earth—is central to human development. Drawing upon studies at the regional and sub-regional level, this review article explores synergies and trade-offs between groundwater development and human development. On one hand, groundwater exploitation may enhance human development. Groundwater’s “untapped potential” related to various aspects of human development involves (a) water supply for irrigation and domestic purposes; (b) climate change adaptation and hydrological resilience; (c) hydrogeological storage of CO_2_; and (d) access to (renewable) energy. On the other hand, human development may come at the expense of quality deterioration or depletion of groundwater. The review concludes that achieving a sound understanding of local groundwater characteristics and human impact on groundwater resources across scales is paramount to implementing the sustainable development goals in an integrated manner.

## Introduction

Groundwater is the most abundant source of fresh water on earth and crucial to life. It is the resource hidden in the pores and cracks underground, after percolating from the earth’s surface or having been trapped due to sedimentation or volcanic activity (Fetter 2001; Fitts 2012). Groundwater is not only the primary source of drinking water for half of the world’s population, but also sustains ecosystems in providing water, nutrients and a relatively stable temperature (Kløve et al. 2011). Humans may rely on such groundwater-related ecosystems for food and energy production, health, and recreation (Machard de Gramont et al. 2011). For example, groundwater is used to irrigate nearly 100 million hectares of arable land and accounts for over 40% of global consumptive water use in irrigation (Siebert et al. 2010). For these reasons, groundwater is intrinsically linked to various aspects of human development, including poverty eradication (e.g. Moench 2003).

Overall, the economic benefits of abstracting groundwater exceed those of surface water per unit volume (Burke and Moench 2000). Groundwater generally moves slowly—at speeds between 0.01 and 10 m per day under natural conditions. The amount of time groundwater spends in storage (i.e. residence times) can range from tens to thousands of years (Foster et al. 2013; Gleeson et al. 2012). Overlying geological formations protect groundwater from climatic fluctuations and pollution. Because of these properties, groundwater is a readily available, relatively reliable source of water that generally requires little treatment (Burke and Moench 2000; Haddad et al. 2000).

Over the course of merely half a century, advances in hydrogeological knowledge, drilling and pumping technology, and rural electrification induced rapid intensification of groundwater exploitation across the world (Foster et al. 2013). Groundwater is the most extracted raw material in the world; its global withdrawal rate of 800–1000 km^3^/year exceeds oil’s by a factor of 20 (Jarvis 2012; Margat and van der Gun 2013). Meanwhile, the demand for fresh water continues to increase worldwide—driven by global population growth, the expansion of irrigated agriculture, and economic development (Wada et al. 2010; Siebert et al. 2010). This increasing demand is largely met by groundwater, especially in those regions that frequently cope with surface water stress (Wada et al. 2010).

Considering the essential role of groundwater in various aspects of human development, we argue that taking a groundwater-inclusive perspective to international development is paramount. The sustainable development goals (SDGs) reflect an integrated, inclusive approach to international development with the aim of promoting increased human well-being, social justice, and environmental sustainability—sometimes called ‘nexus approach’ (Griggs et al. 2013, 2014; Boas et al. 2016; Stafford-Smith et al. 2016; Gupta and Vegelin 2016). Several scholars pointed out that conflicts may arise in the interaction between goals or targets (Griggs et al. 2014; Stafford-Smith et al. 2016; Kim 2016). As put by Kim (2016, p. 17), “even in an ideal world where all the SDG targets are met individually, the outcome may not necessarily be the desired state of sustainable development” as long as there is no mechanism to enhance internal synergies or diminish trade-offs.

During the negotiation of the SDGs, stakeholder groups had issued recommendations as to advocate for the importance of groundwater (Conti 2015). Groundwater was embodied in the final declaration that was adopted on 25 September 2015.

This declaration entails various grounds for human development through groundwater *exploitation*, including the dedicated water goal (Goal 6)—“Ensure availability and sustainable management of water and sanitation for all”. On the other hand, Target 6.6 explicitly calls for groundwater *protection*: “By 2020, protect and restore water-related ecosystems, including mountains, forests, wetlands, aquifers and lakes” (UNGA 2015, p. 18). The conditions for reconciliation of human development and groundwater sustainability have not yet been studied in a systematic manner.

This article poses the question: *How could an improved understanding of potential synergies and trade*-*offs between groundwater development and human development inform the implementation of the sustainable development goals?* This article identifies potential synergies and trade-offs between various aspects of human development and groundwater sustainability, i.e. areas where the first may be beneficial or detrimental to the latter. Human development is understood as the processes by which people can fulfil their basic needs and expand choices and capabilities to lead a qualitative life and to develop their full potential (Hirai 2017; UNDP 2015). Groundwater sustainability is defined as the continued availability of groundwater of sufficient quality and quantity for ecosystem functions and for future generations.

This article reviews studies on the relationship between human development and groundwater development on the global, regional, and sub-regional level. To identify potential synergies, the first part focuses on the importance of groundwater in enhancing key aspects of human development, namely food security, access to drinking water and sanitation, access to renewable energy and resilience in the face of extreme weather events (“Synergies: the contribution of groundwater development to human development” section). Regarding these synergies, the literature selected for review primarily consists of case studies on the benefits of groundwater development to the selected aspects of human development. To identify potential trade-offs, the second part links human development to groundwater unsustainability in terms of both depletion and pollution (“Trade-offs: groundwater resources under pressure due to human development” section). This part relies on a review of literature on the relationships between groundwater sustainability and those aspects of human development outlined above, including case studies and global (water balance) analyses.

Rather than an exhaustive overview of relationships between the SDGs, the review culminates into key policy recommendations to enhance (a) key opportunities of achieving the goals through groundwater development and to address (b) potential risks that the implementation of the goals poses to the sustainability of groundwater resources.

## Synergies: the contribution of groundwater development to human development

### Food security

The SDG agenda includes achieving food security—including the universal eradication of hunger—by means of increased access to food, improved agricultural productivity, and sustainable food production systems (Goal 2 and targets 2.1, 2.3 and 2.4, respectively) (UNGA 2015). Irrigation is directly linked to one key aspects of food security, i.e. agricultural productivity. Critical water needs during specific points in plants’ growth cycles have been well documented for many crops, where substantial yield losses may result from temporary moisture stress (Dingman 2002; Moench et al. 2003). Thus, irrigation helps to avoid situations where water levels fall below critical water needs.

Due to the “inherent flexibility of groundwater (on demand, just in time)”, use of groundwater for irrigation can be associated with the aversion of harvest failure risks and may thus amplify agricultural productivity gains commonly associated with irrigation (Moench et al. 2003, p. 7). Increased reliability of water supply can also generate capital for investments in other production inputs, such as fertilisers or harvesting technologies, which further increase yields (Burke 2002; Moench et al. 2003). Access to groundwater may thus facilitate a “move out of poverty” and enable subsistence farmers to develop alternative livelihoods (Moench 2003, p. 441).

Some regions of the world have room for further development of groundwater to fully reap the economic benefits associated with its use (Margat and van der Gun 2013). Rain-fed agriculture is the main source of food for the better part of the population of Sub-Saharan Africa, which yields cereals and roots of “limited nutritional content and low market value” (Domenech 2015, p. 24). Meanwhile, the potential for groundwater development in this region is considerable. The total area that can be irrigated with renewable groundwater in Sub-Saharan Africa is estimated between approximately 20 and 50 times the present groundwater-irrigated area (Altchenko and Villholth 2015).

Annual consumptive use of groundwater in food production is estimated at 545 km^3^, but groundwater’s share in irrigation varies strongly among continents, regions, sub-regions (Siebert et al. 2010). Further, global assessments tend to rely on datasets that do not include small-scale groundwater irrigation. This would imply that irrigated area and associated yield may well be underreported (Moench et al. 2003; Liu et al. 2009). Thus, one must be careful as to not overstate remaining potential for groundwater irrigation on a global level.

The contribution of groundwater to eradicating hunger and enhance food security through optimising its irrigation potential is subject to generic boundary conditions, irrespective of the source of irrigation water. First and foremost, the crops must be intended for consumption (as opposed to, e.g. the generation of bio-energy). Secondly, local hydrogeological conditions must be taken into account when selecting the type of crop and the irrigation technique, since agricultural productivity can vary depending on a constellation of factors such as the quality of the resource base and water use efficiency (Zwart and Bastiaanssen 2004; Mohammed and Mazahreh 2003). Thirdly, energy must be available at an affordable cost, since this conditions the potential of groundwater exploration to transform irrigation from a water-restricted to an energy-restricted activity. For example, Abramson et al. (2014) estimated that realistic energy price fluctuations may produce up to 30% fluctuations of total costs for both diesel and solar powered pumping schemes.

A final note of consideration is that at the heart of the concept of food security is populations’ ability to *purchase* food, rather than to *produce* it (Lopez-Gunn and Llamas 2008). As put by Moench et al. (2003, p. 5), the equation linking water security and food security “is a function of the interaction between water access, production economics and the wider network of entitlements that water users and others have within society”. Thus, it can be argued that the full realisation of groundwater’s potential in enhancing food security requires a system of rights and entitlements that ensures sustainable, equitable access to and allocation of groundwater, a notion embodied in Target 1.4.

### Drinking water and sanitation

Access to safe drinking water, sanitation and hygiene is not only fundamental to human survival and quality of life, but also ties into broader aspects of human development including dignity and gender equality (Hutton and Chase 2016). The Millennium Development Goals, the international development agenda that preceded the SDGs, was focused towards improving access to drinking water and sanitation services. SDG 6 reinstates the ambition of achieving universal access to safe water, adequate sanitation and hygiene and the elimination of open defecation (Target 6.1 and 6.2). Furthermore, the overall linguistic and ideological framing of the SDGs emphasises the notion of inclusiveness—ensuring access for women and marginalised populations (Hutton and Chase 2016).

Coverage of drinking water and sanitation services has been well monitored under the Millennium Development Goals (Hutton and Chase 2016). Approximately 2.6 billion people reportedly gained access to improved drinking water between 1990 and 2015, yet regional and urban–rural disparities remain. Of those 768 million people who still used unimproved sources in 2011, over 80% lives in rural areas (United Nations 2013). Groundwater development can qualify as a means to provide “improved” access to drinking water; boreholes, tube wells, and protected dug wells all are considered as such in the monitoring strategy for the drinking water indicator of Goal 6 (IAEG-SDG 2015, p. 2).

Groundwater of good natural quality is an excellent source of drinking water. It is overlain by layers of rock, soil or sediment, which filter particles, (pathogenic) microorganisms and insoluble chemical constituents from any percolating (rain-)water (Howard et al. 2006). From a climatic perspective, groundwater’s importance is particularly pronounced in arid and semi-arid areas where surface water is scarce. From a socio-geographic perspective, groundwater is of paramount importance to rural populations that are located away from surface water and piped infrastructure. The “ubiquitous hand-pump-fitted-borehole” is already estimated to serve 1.3 billion rural dwellers (Abramson et al. 2014). Yet groundwater is also of increasing importance in (sub-) urban supply, currently satisfying the domestic demand of an estimated number of 1.5 billion city dwellers. ‘Untapped’ groundwater potential has not been estimated on a global level, but the deployment of manual drilling technologies is estimated to benefit another 90 million people in Sub-Saharan Africa alone (Carter and Bevan 2008). This illustrates that improving access to groundwater may be transformative to the low-income populations of rural areas who currently lack the means to invest in basic infrastructure.

### Climate change mitigation and adaptation

The sense of urgency around climate change has been growing in the global community. The SDG agenda urges to take action to mitigate climate change and its socio-economic impacts (Goal 13) and to ensure universal access to affordable, reliable, sustainable and modern energy (Goal 7). These goals embody the notion that climate change and the impending exhaustion of fossil fuel resources conjunctively necessitate a transition to renewable energy (Yillia 2016). Studies of the impacts of climate change on groundwater resources are plentiful. For example, global climate models have linked oceanic and atmospheric circulation of carbon with groundwater levels, recharge and salinisation (see also “Kaleidoscope of human impact on groundwater resources” section). On the other hand, groundwater presents opportunities with respect to both climate change mitigation and adaptation.

#### Climate change mitigation

To mitigate climate change, carbon sequestration and use of renewable energy resources can reduce atmospheric emission of carbon dioxide (CO_2_). Carbon sequestration in groundwater resources is a growing field of research, particularly concerning deep saline aquifers (e.g. Eccles et al. 2009). Laboratory incubations have found that the water pH decline, associated with CO_2_ infiltration under oxidizing conditions, can increase concentrations of some alkaline earth elements and heavy metals by over two orders of magnitude, while a share of the samples also saw an increase in “[p]otentially dangerous uranium and barium” (Little and Jackson 2010). However, much remains to be understood regarding the physical processes that control potential leakage of CO_2_ and CH_4_ through wells or along faults and fractures (Damen et al. 2006).

Some groundwater resources have the potential for recovery of renewable energy. Benefits related to direct use of geothermal heat include spas and municipal heating in Iceland, France, China and Turkey (Fridleifsson 2001). Worldwide electric geothermal energy capacity amounted to 12 GW in 2013 (IEA 2015), making up for approximately 0.3% of the total capacity. Direct usage of heat was in the same order of magnitude. Besides groundwater dominated systems, these International Energy Association estimates presumably include conductive systems of rock or magma. Moreover, cavernous groundwater resources with fast moving flow (also called karst aquifers) have a potential for hydropower development. Underground dams were constructed in China, Bosnia and Herzegovina, Indonesia, Iraq and Japan (Fiorillo 2015; WWAP 2014). Hydroelectric production reached 1128 GW in 2013, i.e. 16% of the total, but the share of groundwater systems is likely dwarfed by hydropower in rivers and lakes.

Geothermal energy and hydropower are among the most rapidly developed sources of energy (IEA 2015). Fridleifsson et al. (2008, p. 61) concluded that “ample opportunities” to further develop geothermal potential remained. Global estimates of total geothermal potential range between a minimum of 35–70 GW and a maximum of 140 GW (Fridleifsson et al. 2008), while potential for further development of hydropower in (karst) aquifers has never been systematically researched. While the contribution of energy recovery from groundwater to the global energy mix is likely to remain modest, these sources of renewable energy can be significant on the national or regional level.

#### Climate change adaptation

Groundwater is considered a natural buffer against climate variability (Green et al. 2011; Kløve et al. 2014). Development of groundwater resources and managed recharge can support adaptation to variable precipitation and evapotranspiration patterns. Enhancing groundwater’s natural buffering capacity is to harvest and store water surpluses in the subsurface for controlled release whenever needed. This dual capture-storage function is in essence the same as for dams and similar hydraulic infrastructure, but managed groundwater recharge has the additional advantages of economic viability for smaller storage capacities and low susceptibility to evaporation (Dillon 2005).

In addition to enhancing water security in the dry season, managed groundwater recharge can be applied to episodic flooding events and it is increasingly being considered as a means of recycling urban stormwater (Pavelic et al. 2012). Cities and other settlements are characterised by high concentrations of social and economic capital, which implies that any natural disaster may come at high costs in terms of both loss of life and economic damage (Lall and Deichmann 2012). The deployment of artificial recharge techniques may thus reduce the costs related to water-related disasters. Further, case studies suggest that managed aquifer recharge has the potential to allow for increased agricultural productivity, reduced impacts of floods, and increased water security (Vrba and Renaud 2016). Multi-criteria decision support tools for site selection of managed groundwater recharge are the subject of on-going development (e.g. Rahman et al. 2012). If coupled with spatially explicit groundwater data, such tools may culminate in the development of potential maps.

## Trade-offs: groundwater resources under pressure due to human development

### Depletion and quality deterioration

Depletion and quality deterioration comprise two distinct yet interrelated issues. Deterioration of groundwater quality is often associated with pollution, but it may the result of over-abstraction.

#### Depletion

In view of increasing abstraction rates, dropping groundwater tables are of growing concern. In 2012, Gleeson, Wada, Bierkens and Van Beek estimated that the total infiltration area required to sustain both groundwater consumption and groundwater-dependent ecosystem services (i.e. the “groundwater footprint”) was roughly 3.5 times the surface area of the world’s aquifers. Groundwater depletion is geographically uneven, but pressure on the capacity of groundwater resources to supply freshwater for human and environmental needs is an issue with global ramifications (Gleeson et al. 2010).

Roughly 1.7 billion people live in geographical areas where groundwater depletion is prevalent, defined as groundwater abstraction in excess of recharge over an extensive area and for a prolonged period of time (Gleeson et al. 2012, 2010). Arid and semi-arid regions are particularly prone to groundwater depletion due to overexploitation because a common response to drought is for people to rely more heavily on groundwater (Famiglietti 2014; Gleeson et al. 2010). Due to its nature as a hidden resource, a groundwater reservoir could be gradually depleted before serious impacts are felt (Dingman 2002).

The impact of groundwater depletion may extend “well beyond decreasing fresh water availability” (Famiglietti 2014, p. 946). Groundwater dependent ecosystems such as springs and wetlands may deteriorate, which in turn affects human populations that rely on various ecosystem services (Kløve et al. 2011). Other impacts include land surface subsidence, sea level rise, seawater intrusion, streamflow diminishment, and regional climate feedbacks (Famiglietti 2014). Continued groundwater depletion is also likely to accelerate desertification, especially along mid-latitude (Famiglietti 2014).

The burden of depletion is largely borne by vulnerable people and marginalised communities, who lack the means to adapt to the dropping groundwater tables, e.g. through digging deeper wells (Famiglietti 2014). A growing body of research reveals the potential connections between (ground-)water scarcity, food security, social conflicts, and human migration patterns (e.g. Metulini et al. 2016; Carter and Parker 2009).

#### Pollution

Anthropogenic pollution of groundwater is distinguished from contamination from other sources, such as soluble minerals that are endemic to the subsurface. Groundwater pollution is “difficult to remediate” due to the “physical inaccessibility and porous structure” of aquifers (Foster et al. 2013, p. 691) and may ultimately threaten both human health and the quality of ecosystems.

Sources of pollution that may pose a threat to human health include so-called ‘point source pollution’ (e.g., seepage from latrines and fecal depositories; landfill leachate; chemical spills at factories or mining sites), and ‘diffuse pollution’ (e.g., storm water runoff from roadways and parking lots; agricultural runoff) (Fitts 2012; Howard 2015; Howard et al. 2006). Leaky pipelines are sometimes perceived as comprising a separate category of ‘line pollution’.

Depending on local groundwater flow patterns, soil-specific properties, and processes on the molecular level, pollutants might disperse rapidly or at “snail’s speed” (Fitts 2012, p. 521). Firstly, pollutants percolate relatively quickly through high-conductivity material such as sand compared to low-conductivity material such as clay. Secondly, some pollutants move little from their source due to adsorption onto soil particles, “while others migrate freely with the flowing pore water” (Fitts 2012, p. 521). Thirdly, chemical reactions along the way can result in disintegration into less harmful substances or the formation of new pollutants, while other pollutants are less reactive (i.e. more ‘persistent’).

Groundwater pollution can take many forms and threaten the health of both human beings and ecosystems in various ways. Seepage of wastewater into groundwater resources, for example, may lead to biotic contamination, posing a risk of transmission of fecal–oral disease to those who rely on these resources for domestic purposes (Howard et al. 2006). Pesticides and herbicides are usually persistent, organic compounds that can migrate great distances. Both leakage from septic systems and agricultural runoff can also lead to nitrification of groundwater (Fitts 2012). Chemical components of personal care products, pharmaceuticals, and industrial compounds comprise a category of emerging drivers of groundwater pollution, where hormones are considered “particularly troubling chemicals” that may disturb aquatic life (Lapworth et al. 2012; Fitts 2012, p. 523).

In addition to pollution, deterioration of the quality of groundwater resources may take the form of salinisation. Wastewater return flows with residues of detergents and washing powders comprise a major driver of salinisation of groundwater, since these dissolved ionic salts are not always removed in conventional treatment. Agricultural return flows may have the same effect, particularly if treated wastewater is applied for irrigation purposes (Vengosh 2013). Salinity intrusion can also be caused or amplified by groundwater over-abstraction (IPCC 2007).

### Kaleidoscope of human impact on groundwater resources

The state of groundwater is subject to (1) resource-specific characteristics, (2) human activity in the catchment area, and (3) socio-economic processes that may transcend the catchment level and even have international implications. Human impact on groundwater is thus the resultant of complex processes on various levels.

Groundwater resources of the world vary widely in terms of various resource characteristics that determine the sustainability of exploitation such as the vulnerability to pollution. Natural water quality depends on the geochemistry of the resource, while the storage-flow relationship determines the proportion of groundwater that has sufficient mobility to influence surface water supplies and aquatic ecosystems through regular replenishment (i.e. ‘active groundwaters’). Negligible recharge of ‘inactive groundwaters’, coupled with intensive use, can result in a virtually permanent depletion of resources, often referred to as ‘groundwater mining’ (Foster and Loucks 2006; Gleeson et al. 2012).

Both the quality and the quantity of groundwater are strongly affected by human activity in the catchment area. Population density and land use are, in turn, influenced by socio-economic factors and governance. Water use efficiency, i.e. the produced output per unit of groundwater, is an important factor for agricultural and industrial use, whilst the per capita water use determines domestic use. Urbanisation has distinctive effects on the water balance and often involves new sources and pathways of pollution. Generally, high urban recharge “tends to counteract the effect of intensive groundwater withdrawals” for unconfined aquifers (Foster et al. 2013, p. 688; Howard 2015). For these reasons, integration of groundwater resource management and land use planning is arguably of paramount importance (Collin and Melloul 2003; Foster et al. 2013).

Pressures on local resources are at least partially attributed to processes that extend the basin level and may even have global ramifications. First, climate change comprises indirect impacts from human activity on groundwater resources. Changes in the water balance may culminate in an overall loss of fresh groundwater resources, despite increased recharge in some localities (Jiménez et al. 2014; Kløve et al. 2014). Changes in groundwater levels and recharge mechanisms may also mobilise new contaminants (Green et al. 2011). Moreover, groundwater resources in coastal areas and small islands will likely become more saline (Holding and Allen 2016; Ranjan et al. 2009). Second, a so-called virtual water trade underlies the international trade in agricultural products and industrial goods (Lopez-Gunn and Llamas 2008; Gupta et al. 2013). Due to both infrastructural and virtual groundwater transfers, impacts of urbanisation may also extend tens of thousands of kilometres beyond cities’ jurisdiction (Hoogesteger and Wester 2015; McDonald et al. 2014).

### Trade-offs in the context of SDG implementation

As stated in the introduction, achieving global sustainable development is a complex endeavour, which involves balancing a wide range of interests and priorities. This section outlines conceivable trade-offs between groundwater protection (Target 6.6) and other goals and targets within the SDG agenda.

#### Food security

Irrigation accounts for the bulk of global water use; approximately 70% of all fresh water withdrawals are appropriated for irrigation, of which 43% is pumped up from the subsurface (Siebert et al. 2010). Many “fossil” groundwater resources with low replenishment are heavily mined for food production. These include the Nubian sandstone aquifer system in northern Africa, the Saq/Ram aquifer system in western Asia, and the Indo-Gangetic plain in southern Asia (Wada et al. 2010, 2012; Ferragina and Canitano 2014). Thus, the relationship between food security and groundwater depletion is an intricate one, particularly in arid regions.

Target 2.4 prescribes the promotion of resilient agricultural practises, but substantiation is needed to avoid groundwater development at the cost of environmental and social inclusiveness. Approximately 11% of groundwater depletion for irrigation is embedded in the international food trade (Dalin et al. 2017). Further, policy instruments for food security and the promotion of biofuels heavily rely on (energy) subsidies (Fraiture et al. 2008): market distortions that are at the heart of the trade-off between groundwater-fed irrigation and depletion. The SDG agenda mandates that “inefficient fossil-fuel subsidies that encourage wasteful consumption” or amount to distortions of international food trade be phased out (Target 12.c), which would diminish this trade-off.

Drinking water and sanitation improving access to water for sanitary uses through groundwater development entails a risk that the quality of the resource is compromised. As discussed in “Depletion and quality deterioration” section, leaching of faecal matter into groundwater resources may pose particular health risks associated with the transmission of water-borne disease, particularly when these resources form a source of drinking water for communities or their livestock.

Such health risks depend on a combination of the technical features of the sanitation solution and geo-hydrological factures (Montgomery and Elimelech 2007). A large share of the effluent from flush toilets, the near universal sanitation solution in the developed world, is discharged directly into the environment (Williams and Overbo 2015). To preserve groundwater resources, alternate means to improve sanitation (such as dry or composting toilets) may therefore be more viable in areas where groundwater tables are shallow or surface water run-off permeates easily into the groundwater (Montgomery and Elimelech 2007).

The inherent inclusivity of the phrasing of Goal 6 minimises the trade-offs between drinking water and sanitation (Targets 6.1 through 6.3) on one hand and groundwater quality (Target 6.6) on the other, as long as the targets of concern are given equal priority in the implementation. The context dependence of risks of faecal contamination of groundwater resources calls for context-appropriate drinking water and sanitation solutions, which take hydrogeological considerations into account.

#### Climate change mitigation and adaptation

Regarding climate change mitigation, context-appropriate use of groundwater’s kinetic and geothermal energy potential may contribute to the global transition to renewable energy, however, modestly. While pumping from large depths may induce seismic activity, there are no imminent trade-offs between geothermal energy recovery from groundwater and other human and environmental uses of the resource. With regard to underground carbon storage, however, caution should be taken to avoid changes in chemical groundwater quality that are detrimental to ecological- or human health.

Careful use of groundwater’s storage capacity can strengthen resilience and adaptive capacity to climate-related hazards and natural disaster. Specifically, it can help mitigate the socio-economic effects of (hydrological) floods or droughts. Nevertheless, quality preservation of groundwater resources likely necessitates the removal of pollutants prior to managed recharge, particularly in urbanised areas (Vanderzalm et al. 2010).

## Conclusions and recommendations

The review of literature above finds that after a few decades of rapid and widespread development of groundwater, opportunities remain to use groundwater’s relative pervasiveness and reliability for the benefit of human development (Table 1). Specifically, this concerns (1) enhancing food security through the optimisation of groundwater irrigation’s potential to increase the reliability of harvests and enable a transition to more nutritious or valuable crops; (2) improving access to groundwater for drinking and sanitation purposes, particularly for geographically or economically marginalised populations; (3) exploring and developing the kinetic and geothermal energy potential of groundwater resources to enhance the level of access to sustainable energy; and (4) where feasible, utilise the unique ‘buffer’ capacity of groundwater to reduce the costs associated with climate-related extremes. On the other hand, human development can come at the expense of groundwater quality or quantity and an understanding those trade-offs is crucial to averting situations where the sustainability of the resource is compromised. Thus, we argue that the implementation of the sustainable development goals must reflect relevant differences between groundwater and surface water resources related to groundwater’s distinctive residence time and relative insensitivity to variation in rainfall and evaporation.

**Table 1:** Potential synergies and trade-offs in groundwater-related implementation of the SDGs

	Potential synergies				Potential trade-offs
	SDG target	(Causal) mechanism	Boundary Conditions	Potential side-effects	SDG target
Food security	Improve small-scale food productivity through productive resources and inputs (**T2.3**)	Relative reliability of groundwater for irrigation averts yield losses during hydrological droughts In response to increased water security, resources may become available for additional productivity inputs Groundwater development thus tends to exacerbate the productivity benefits associated with irrigation Remaining groundwater development potential has not been researched on a global scale, but case studies suggest millions of farmers can benefit	Equitable access to resources and land ownership (**T1.4**) High water use efficiency (**T6.4**) Access to affordable energy for pumping Sustainable food chains and resilient agricultural practises (**T2.4**). Equitable food trade	Eradicate hunger, malnutrition, and child mortality especially among infants (**T2.1, T2.2 and T3.2**) Eradicate extreme poverty (**T1.1**) Increase exports from developing countries (**T17.11**)	Development at the expense of availability in the future (**T6.6**) Development at the expense of availability for water-related ecosystems (**T6.5**) or other aspects of human development
Drinking water and sanitation	Universal access to safe and affordable water for drinking (**T6.1**) and sanitation and hygiene (**T6.2**).	Groundwater of good natural quality is relatively suitable for consumption, due to filtering properties of overlying material (protection from abiotic and microbial pollution) Remaining groundwater development potential has not been researched on a global scale, but case studies suggest millions of rural dweller can benefit	Lower water pollution (**T6.3**), e.g. through ending open defecation (**T6.2**), environmentally sound management of (chemical) waste (**T12.4**)	Eliminate gender disparities in access to education (**T4.5**) and participation in public life (**T5.5**) Reduction of water-borne diseases (**T3.3**) and child mortality (**T3.2**)	Waste water leaching at at the expense of future groundwater quality (**T6.6**)
Climate change mitigation	Substantially increase the share of renewables in the global energy mix (**T7.2**)	Carbon sequestration contributes to lowering atmospheric CO_2_ concentrations. Successful pilot-based deployment of carbon sequestration in groundwater Kinetic or geothermal energy recovery potential considerable on regional level, but modest compared to global energy demand	Potential for energy recovery is subject to hydrogeological and geothermal factors, where geochemical properties determine the viability of the equipment (Zektser and Everett 2004)	Eradicate extreme poverty (**T1.1**)	Development at the cost of groundwater quality for water-dependent ecosystems (**T6.5**) or other human development aspects
Climate change adaptation	Combat desertification, droughts and floods (**T15.3**)	Groundwater resources serve as water reserves during hydrological droughts and can absorb excess storm water to combat (urban) flooding Case studies suggest Managed Aquifer Recharge can have positive effects, but systematic feasibility studies remain lacking	Capacity for participatory, integrated and sustainable human settlement planning (**T11.3**)	Build resilience of the poor to climate-related extreme events and disasters (**T1.5**) and strengthen adaptive capacity to climate-related hazards (**T13.1**) Reduce casualties and economic loss from (water-related) disaster (**T11.5**)	Development at the cost of quantity (when used as buffer) and/or quality (when deploying managed aquifer recharge) in the future (**T6.6**)

We further present the following conclusions and associated policy recommendations. First, the analysis of synergies shows that groundwater’s potential contribution to human development is predicated upon local (hydrogeological and climatological) conditions that determine available quantity, quality and the feasibility of abstraction. As a result, the costs for groundwater development show large variations on both the international and the national level, subject to economic, technical and environmental variables (Abramson et al. 2014). Similarly, potential trade-offs are the product of human impact and the physical vulnerabilities of the groundwater resource. For example, deep aquifers with limited renewability are relatively sensitive to depletion, whereas alluvial aquifers overlain with permeable material are relatively sensitive to quality deterioration.

In light of the heterogeneity of groundwater resources across the world, we argue that achieving a sound understanding of local groundwater characteristics is paramount to implementing the SDG agenda. As such, we recommend bolstering the implementation process to promote increased data gathering and assessments of groundwater development potential for the benefit of various aspects of human development, while taking potential trade-offs into account. At the same time, it is important to understand the processes, including physical and virtual water transfers, which shape human impact on groundwater resources across scales—particularly with respect to the most pressing trade-offs in the context of food security. Second, we conclude that the general lack of capacity with respect to groundwater resources management is a major hindrance to benefiting from synergies and addressing trade-offs between groundwater and human development. As such, we recommend harnessing the “Post-2015 Data Revolution” to gather empirical data on water use, disaggregated by source and to develop capacity for hydrogeological analyses, which will provide the basis for feasibility studies of groundwater development in regions where groundwater is under-utilised. Further, we recommend developing the body of knowledge by increasing awareness of the unique properties of groundwater—intensifying research and developing linkages with well-funded issues accordingly. Specific subjects of further study include the potential of managed aquifer recharge, worldwide, and groundwater in relation to the causes and consequences of rural–urban migration patterns. Since cities will have to accommodate another 1 billion inhabitants over the next two decades, understanding synergies and trade-offs in the context of urban groundwater development will be paramount.
